# Parental Stress, Learned Helplessness, and Perceived Social Support in Mothers of Children with Hearing Loss and Mothers of Typically Developing Children

**DOI:** 10.3390/audiolres15010001

**Published:** 2024-12-25

**Authors:** Usha Shastri, Niya Prakasan, Lakshmi Satheesan, Kaushlendra Kumar, Mohan Kumar Kalaiah

**Affiliations:** Department of Audiology and Speech Language Pathology, Kasturba Medical College Mangalore, Manipal Academy of Higher Education, Manipal 576104, Karnataka, India; usha.shastri@manipal.edu (U.S.);

**Keywords:** parental stress, age of hearing loss identification, hearing aids, cochlear implants, bimodal hearing, age of onset of rehabilitation, socioeconomic status

## Abstract

**Background:** Hearing loss in children can have a detrimental impact on their development, thus lowering the psychological well-being of parents. This study examined the amount of parental stress, learned helplessness, and perceived social support in mothers of children with hearing loss (MCHL) and mothers of typically developing children (MTDC), as well as the relationship between various possible contributing factors to parental stress such as learned helplessness and perceived social support. **Method:** Three questionnaires measured parental stress (Parental Stress Scale; PSS), learned helplessness (Learned Helplessness Scale; LHS), and perceived social support (Perceived Social Support-Friends PSS-Fr and Perceived Social Support-Family PSS-Fa Scale) in 100 MCHL and 90 MTDC. All mothers had normal hearing and children aged 1 to 12 years. Mothers of children with any degree of bilateral sensorineural hearing loss, using hearing aids and/or cochlear implants, were included in the MCHL group. Children with hearing loss did not have any additional disabilities. **Results:** Parental stress and learned helplessness were not significantly different between the groups. The perceived social support was significantly lower in MCHL as compared to MTDC. Pearson correlation analysis with pooled data from both groups showed a small but significant negative correlation between parental stress and perceived social support. A moderately significant positive correlation existed between learned helplessness and parental stress. The relationship between learned helplessness and perceived social support was negative, being small but significant. **Conclusions:** The study findings indicate that parental stress was similar in both groups. As perceived social support increased, parental stress and learned helplessness decreased. Thus, the present study findings indicate the need for developing greater social support for MCHL.

## 1. Introduction

Parenting is a fundamental aspect of the survival of every living being and is central to the success of the human race [1]. Parenting a child with disabilities can be a rewarding experience filled with joy and fulfillment, but it poses its own set of challenges and caregiving burdens [2]. Prior research has highlighted the crucial impact of parenting, where parents who have children with disabilities experience higher levels of dissatisfaction and weak bonding with their children when compared to parents of children without disabilities [3,4]. Parental stress is defined as a specific type of stress that occurs when the demands of parenting surpass the resources available to a parent, leading to a feeling of being overwhelmed [5]. This arises because society does not understand and accept their children, and thereby parents often emphasize the negative aspects of disability [6]. Learned helplessness is a condition that occurs when people assign negative results to their internal, stable, and global factors, leading them to believe that they have no control over their situation [7]. Consequently, such individuals do not try to change even when such opportunities are available in the future [8]. Rehabilitation for children with disabilities requires long-term commitment from the parents and the improvements may not be quickly seen. Hence, it is possible that they feel that their challenges are not going to reduce no matter what they do. Learned helplessness and stress are more likely to occur when an individual’s coping resources are outweighed by the demands of society. These demands are found to be higher for parents nurturing a child with disabilities [9].

Perceived social support is the sense of emotional fulfillment, respect, support, and being understood within one’s society [10]. It is mainly derived from personal relationships, primarily with family members, friends, and neighbors, offering various forms of help, including emotional support, practical aid, advice, companionship, and respect [11]. Social relationships with friends and family members help to maintain balance, stability, and cohesion in the family and support all family members in tackling challenges, fulfilling their roles, and ensuring positive interactions with one another [12]. Researchers have found social networking to be a potential buffer against stress and that it has improved the quality of life of many parents and children with disabilities [13]. Studies indicate that greater perceived social support is associated with reduced experience of stress in parents of children with hearing loss [14,15].

Among different impairments, hearing loss in children is an important cause of parental stress [6,14,15,16]. Congenital hearing loss can have long-term developmental implications for communicative, linguistic, behavioral, academic, and socioemotional functions. It results in distinctive and long-term challenges for parents in terms of increased educational, medical/audiological care, and communication difficulties [15]. Hence, congenital hearing loss is thought to have a detrimental impact on a child’s development, thus lowering the psychological well-being of parents [17].

Many parents of children with hearing loss (CHL) worry that their children will receive fewer opportunities in their lives [18]. The mothers of children with hearing loss (MCHL) are more stressed and have a high rate of psychological and mental health problems such as depression and anxiety [16]. Stress can also be in response to the diagnosis of their child, increased financial strain, difficult communication or interaction with their children, feelings of isolation, and adjusting to the needs of their children with hearing loss [19]. According to Park and Yoon [20], the feelings of helplessness regarding how to raise their children may be exacerbated by the lack of sensitivity from hospitals and doctors toward the parents of CHL. According to international studies, hearing loss in children can accelerate negative self-attitudes, thereby causing social isolation for their mothers. This situation renders mothers more prone to experiencing stress and various disorders [21]. However, study findings by Asberg et al. [14] and Chang et al. [22] reported no difference in stress levels between parents of children with normal hearing and parents of CHL.

In terms of social support, initial studies found that MCHL experienced higher levels of stress and were less satisfied with their social support compared to mothers of children with normal hearing [15,23]. According to Quittner et al. [23], MCHL had smaller social networks, less frequent contact with family and friends, and less social support overall. Review studies also highlight the importance of social support for parents of children with hearing loss [24]. Recent studies indicate that greater levels of parenting stress are associated with greater social support needs [25,26]. Thus, it is apparent that over a period of years, even with the availability of hearing technologies like cochlear implants, the requirement for social support has not changed.

Several factors contribute to the stress experienced by parents of children who use amplification devices. These factors include the age of identification and amplification, the degree of hearing loss, low family income, the high cost of amplification devices, inadequate speech perception and production, inappropriate behavior and characteristics of the child, poor socialization among peers, and unrealistic expectations regarding their child’s communication and education skills [27,28,29,30]. Notably, aspects like the poor social–emotional skills and language abilities of CHL significantly elevate stress levels among these parents [31], and it is also possible that increased parental stress results in difficulties in the development of the social–emotional and language skills of a CHL. Studies have compared parental stress levels between parents of children with hearing aids and parents of children with cochlear implants (CI). Some studies reported lower stress levels among parents of children with CI [28,32], whereas other findings reported heightened levels of psychological distress in MCHL with CI [33]. Another study by Asberg et al. [14] found no difference in stress levels between parents of children with CI or hearing aids.

There are a few concomitant factors affecting parental stress [34]. Some of them include reduced quality of life at the time of diagnosis, reduced family support, reduced social support, socio-emotional concerns, lower acceptance by other family members and society, lower education, lack of time for oneself, lower self-competency, and communication barriers with the child. According to Continisio et al. [6], a low level of education can be a risk factor for parental stress. However, no correlation could be observed between the age of the mother or the child and parental stress. Another important factor for parental stress is lower income [19]. Sarant et al. [35] reported a lack of social support as the primary cause of parental stress.

Hearing loss is a condition that requires long-term persistent effort from the parents for rehabilitation of their CHL. Hence, the parents might give up on their rehabilitation efforts if they do not receive adequate support from their environment. In addition, doing too much for their child or over-functioning for them is likely to result in learned helplessness in parents. Thus, mothers of children with hearing loss may acquire a varied degree of stress and helplessness, especially if the perceived social support is low. This, in turn, may affect the motivation of parents in working towards the rehabilitation of a child with hearing loss. Hence, there is a need to look at the effects of perceived social support and learned helplessness on parental stress in MCHL. Many of the recent studies have focused on parental stress and children with hearing loss [25,27,34]. To the best of our knowledge, learned helplessness has not been studied in MCHL. Hence, the present study aimed to examine the amount of parental stress, learned helplessness, and perceived social support in MCHL and mothers of typically developing children (MTDC). This study also examined the interplay between various factors related to the child, family, and socioeconomic status and parental stress in MCHL and MTDC. It was hypothesized that lower levels of perceived social support and greater levels of learned helplessness might increase parental stress.

## 2. Materials and Methods

This cross-sectional study was approved by the Institutional Ethics Committee and was initiated after obtaining informed consent from the participants.

### 2.1. Participants

A total of 100 MCHL and 90 MTDC participated in this study. Mothers of children aged 1 to 12 years identified as having any degree of bilateral sensorineural hearing loss and who are using hearing aids (HAs) and/or CIs were eligible for inclusion in the MCHL group. Mothers of children with conductive hearing loss, unilateral hearing loss, additional disabilities, and not using any amplification device were excluded from the study. The mean age of identification of hearing loss was 9.2 months (SD = 9.43, median = 6.5 months), and the mean age of providing an amplification device was 21.25 months (SD = 15.79, median = 19 months). A total of 81% (N = 81) of the MCHL reported that their children had bilateral severe to profound hearing loss in both ears. The remaining MCHL reported a lesser degree of hearing loss ranging from a minimal to a severe degree of hearing loss [36]. Regarding the use of amplification devices, 49% (N = 49) of the children were using HA in one ear and a CI in the other ear, 28% (N = 28) of children used a unilateral CI, and the remaining 23% (N = 23) children used only HAs as their amplification device. All but four MCHLs reported that they communicate with their children verbally. These four mothers reported that they mainly communicated with their children using gestures.

The MTDC group included mothers of children aged between 1 and 12 years with no disabilities. None of the mothers in either group reported any difficulties in hearing. All of the mothers had a minimum educational level of at least 10th grade. In MCHL, 91% of the mothers were homemakers, while 62.22% of the MTDC were homemakers. Table 1 provides the age, number of children, birth order, and type of family of typically developing children (TDC) and CHL. Table 2 provides the education and occupation of mothers and fathers in both groups.

Certain statistical analyses were performed to assess the similarity between the two groups. The age of the children did not follow a normal distribution (*p* < 0.05) according to the Shapiro–Wilk test of normality. Hence, the Mann–Whitney U test was carried out to assess the significance of the age difference between the groups. The results revealed that TDC were significantly younger than CHL (z = 6283, *p* < 0.001). The chi-square test of association for having one child or more than one child with the groups showed no significant association [χ^2^ (1) = 2.16, *p* = 0.14]. Similarly, family type (nuclear vs. joint) did not show a significant association with the groups [χ^2^ (1) = 2.55, *p* = 0.11]. The education level of parents was categorized into two levels, graduates and above vs. plus 2 and below, for the purpose of statistical analysis. The chi-square test of association showed a significant association, such that there were more mothers who had graduate education in the MTDC group than the MCHL group [χ^2^ (1) = 13.36, *p* < 0.001]. The chi-square test of association also showed a significant association such that there were more fathers who had graduate education in the MTDC group than the MCHL group [χ^2^ (1) = 11.59, *p* < 0.001]. Mother’s occupation was also significantly associated with groups [χ^2^ (1) = 22.40, *p* < 0.001], such that the MCHL group had more homemakers than the MTDC group. All fathers, except four in the MCHL group, were working. Thus, the groups were similar in terms of family characteristics, such as the number of children and family type, but different in socioeconomic status and age.

### 2.2. Materials

The following three questionnaires were used in this study.

Parental stress scale (Berry and Jones [37]): This scale consists of 18 questions designed specifically to quantify the stress level of parents by considering positive (emotional benefits, personal growth) and negative (resource demands, constraints) aspects of parenting. Respondents agree or disagree in terms of their relationship with their child or children. Their responses are rated on a 5-point rating scale as strongly disagree, disagree, undecided, agree, and strongly agree. The overall possible scores on the scale range from 18 to 90. The greater the score, the higher the level of parental stress that has been measured. Berry and Jones [37] and Zelman and Ferro [38] reported good internal consistency coefficients with Cronbach alpha values ranging from 0.83 to 0.86. In addition, the test–retest correlation was reported to be 0.81 [36].

Learned helplessness scale (LHS; Quinless and Nelson [39]): This is a 20-item self-report questionnaire developed to measure the amount of learned helplessness experienced by the individual. The participant have to provide a rating on a 4-point Likert scale corresponding to strongly agree, agree, disagree, and strongly disagree based on how closely they agreed or disagreed with each item’s description of themselves or their feelings about themselves. The LHS scores can range from 20 to 80, with higher scores suggesting greater learned helplessness. Quinless and Nelson [39] reported a good internal consistency coefficient with Cronbach alpha of 0.85.

Perceived social support-friends (PSS-Fr) and perceived social support-family (PSS-Fa) scale (Procidano and Heller [40]): Both PSS-Fr and PSS-Fa consist of 20 items with declarative statements to which the person responds “Yes”, “No”, or “Don’t know” to the feelings and experiences that most people have in their relationships with friends and family at some point. The scores of each scale are added separately, and finally, the scores of both scales are added together. The score range is from 0 to 40, with a higher score indicating higher perceived social support. The internal consistency of PSS-Fr and PSS-Fa was reported to be high, with Cronbach’s alpha of 0.88 and 0.90, respectively [40].

### 2.3. Procedure

The questionnaires were incorporated into a Google Form. The links to the questionnaires were distributed via email among clinicians and institutions that provide auditory rehabilitation services for CHL and were requested to distribute them to MCHL. Two surveys were created, specifically one for the MCHL and the other for the MTDC. Mothers who consented to participate proceeded to fill out the form. The participants read the questionnaires and filled out the responses online.

The survey began with obtaining the demographic details of the participants following the audiological history of the child, which involved details regarding the type and the degree of their child’s hearing loss, use of amplification devices, age at which they started using the device, and the duration of usage of the device. Information about the speech and language therapy undergone by the child was also collected. The questions regarding hearing loss and its details were excluded from the MTDC group survey. The survey form was divided into sections appropriately so that the respondents were able to answer the questions applicable to them.

A brief overview of the questionnaire was provided at the beginning of each questionnaire section. The questionnaire for MCHL took about 30–35 min to complete, whereas the questionnaire for MTDC took about 20–25 min. All the questions were marked as required so that the respondent did not miss out any answers. Participation in the study was anonymous, voluntary, and no specific benefits were given.

### 2.4. Statistical Analysis

The obtained data were tabulated, the total scores for each questionnaire were noted, and descriptive statistics were analyzed. The Shapiro–Wilk test of normality was administered to check for the normal distribution of the numeric data. The results revealed that the scores for all three questionnaires were normally distributed (*p* > 0.05). Hence, a decision was made to run a parametric independent samples t-test to understand the difference between the groups in these questionnaires. These analyses were performed using SPSS software version 29 [41].

## 3. Results

### 3.1. Comparison of the Parental Stress, Learned Helplessness, and Perceived Social Support Between MCHL and MTDC

Table 3 shows the mean, standard deviation, and range of parental stress, learned helplessness, and perceived social support in MCHL and MTDC. From Table 3, it can be observed that PSS-Fr, PSS-Fa, and PSS-Total were less in MCHL than in MTDC. Independent-sample *t*-tests for each of these scores between the groups revealed that the amount of parental stress and learned helplessness was not significantly different among MTDC and MCHL. However, the amount of perceived social support was significantly greater in the MTDC group as opposed to the MCHL group. This was true for PSS-Fr, PSS-Fa, and PSS-Total. The statistical values of these tests are also provided in Table 3.

### 3.2. Relationship Between Parental Stress, Learned Helplessness and Perceived Social Support

The Pearson correlation coefficient was calculated for the scores of questionnaires measuring parental stress, learned helplessness, and perceived social support. Data from both groups were pooled in this analysis (N = 190). The results show that there was a small but significant negative correlation between parental stress and perceived social support (r = −0.15, *p* = 0.04). Thus, as the perceived social support increased, parental stress decreased. Furthermore, a moderately significant positive correlation existed between learned helplessness and parental stress (r = 0.41, *p* < 0.001). This indicated that as the learned helplessness increased, parental stress also increased. The relationship between learned helplessness and perceived social support was also small but significant (r = −0.26, *p* < 0.001), such that as the perceived social support increased, the learned helplessness decreased.

## 4. Discussion

### 4.1. Parental Stress, Learned Helplessness, and Perceived Social Support Between MCHL and MTDC

The findings of the present study revealed that the MCHL group experienced levels of parental stress like those of the MTDC group. This finding supports the results of other studies in the literature [4,14,35,42]. This could be due to the possibility of adopting an appropriate and effective rehabilitation method and the support received by mothers/parents in specialized services for CHL. This support received is conducive to reducing, among other things, anxiety and depression when raising a child with hearing loss [43]. In contrast, some studies have provided contradictory results to that of the present study [6,14,15,16,44]. Some of these discrepancies could be attributed to the different sources of parental stress measured using different scales. For example, Van Driessche et al. [44] used the Zarit Burden Scale which addressed the emotional, physical, and financial burden experienced by caregivers. The analysis of the scale revealed that the stress experienced was high among the parents of CHL.

Many parents are likely to develop learned helplessness when they do too much for their child or over-function for them, meaning that that their child does not develop competence or mastery later in life. Doing too much for their child later makes it harder for them to stop. We expected that this behavior would be more common in MCHL than in MTDC, as CHL require more support from their parents, especially in the initial stages of development. However, the results of the present study found that the level of learned helplessness behavior between the MCHL and MTDC groups was not significantly different. Recent advancements in the rehabilitative field and the availability of the appropriate services might have provided more courage for these mothers to face the challenges of raising their CHL [43]. Previous researchers had examined the problem-solving ability of MCHL and found that it was associated with the adaptability of their child to the environment, a developed sense of coherence, and the satisfaction of mothers regarding the language development of their child [45,46]. Also, utilizing the available resources and acquiring new resources would promote the coping behavior of the parents and lead to better functioning and adjustment to overcome the challenges of raising a CHL [47]. Since all the CHL in the present study who were below 5 years were currently attending speech and language therapy, it is possible that these mothers had access to new resources which helped to promote coping behaviors. In addition, except for four, all MCHL reported that they communicate with their child verbally, which may have led to satisfaction and better functioning among these parents. The mothers of the other four CHL reported that they use more gestures while communicating with their children.

The perceived social support from friends and family and the total perceived social support were significantly lower in the MCHL group as compared to the MTDC group. This is in agreement with Marie et al. [25], who stated that MCHL experience less support, especially with less support from friends and family. Quittner et al. [23] reported that the MCHL subsequently relied more on health professionals in seeking help rather than their family and friends. They also reported that these mothers were found to be withdrawn from maintaining relationships with others. This was because they feel embarrassed to maintain or access a proper social network due to the impairment of their child and require more time to spend with their children. Thus, they are withdrawn from the community and other social activities and events [48]. One of the other reasons for this perceived negativity is the inappropriate behavior and characteristics of their child, which exert pressure on parents to seek out help from others, especially family and friends [49]. It is to be noted here that the reduced social support experienced by parents of CHL has not changed over the last 30 years. Also, it is important to note that parents require support from their family, friends, and acquaintances whether the child has hearing loss [50] or other disabilities [51].

Cultural factors are an important factor to be considered while attempting to generalize the findings from this study. Studies have reported that different social, cultural, and community factors are linked to the parental stress experienced by parents [52,53], especially when raising a child who has a disability [54,55]. Cultural differences are also present in the utility of perceived social support in parenting [56]. A similarity between the present study’s findings and those in studies from the literature across cultures is the need for perceived social support, which is not sufficiently received by MCHL.

### 4.2. Relationship Between Parental Stress, Learned Helplessness, and Perceived Social Support

Many studies have found that increased social networking can result in reduced stress and thus improved psychological well-being in MCHL [24,44,57]. Thus, there is a need to provide social support for MCHL, leading to better motivation regarding the rehabilitation of the CHL. The findings of the present study indicate that there was a negative correlation observed between stress experienced by both groups and perceived social support. This is similar to the findings of Pipp-Siegel et al. [19], Asberg et al. [14], and Dirks et al. [26], who reported that parents with greater perceived social support experienced lesser stress. The negative correlation between parental stress and perceived social support could be due to the fact that parents with greater stress tend to seek out more support in terms of information about their child’s hearing loss, their functioning, and opportunities to connect with other parents facing similar challenges [33].

Our results indicate a significant positive correlation between parental stress and learned helplessness. This indicates that as the parental stress increased, learned helplessness also increased. Previous studies have not directly investigated the relationship between parental stress and learned helplessness. Several additional factors might play a role in the relationship between stress and learned helplessness. It is possible that MCHL actively try to obtain resources that could help them to cope with the challenges they face during the raising of a child with a hearing impairment. Furthermore, the amount of parental stress in mothers might also depend on the specific type of impairment (hearing impairment vs. other impairments) that the child is living with [58].

The current study showed a small but significant negative correlation between perceived social support and learned helplessness in both groups. This indicates that as the perceived social support decreased, the learned helplessness increased. To the best of our knowledge, such a relationship has not been investigated in parents of CHL or those with any other disability. Hence, evidence from the literature on a different population was sought. Learned helplessness experienced by individuals following acute myocardial infarction indicated a negative correlation with perceived social support [59]. This study found that those individuals who received greater support from society experienced reduced learned helplessness.

### 4.3. Influence of Various Variables to Parental Stress

Several factors may be related to parental stress, namely child-related factors, mother-related factors, family-related factors, and the socioeconomic status of parents. Research suggests that higher education of parents can lower stress levels [60]. Lower income also contributes significantly to parental stress [19,61,62]. Another research finding by Gross reported that the mothers of third-born CHL experienced the highest parenting stress [63]. Some findings with respect to child-related factors reported that parental stress increases with an increase in the child’s age [64,65], while some reported no correlation between child’s age and parental stress [14,66]. Several other concerns of parents include the age of activation of the HA or CI device, the degree of hearing loss, reduced affordability of the amplification device, poor outcomes related to the device, inadequate speech perception and production, poor socialization, high expectations regarding the communication and educational skills of their child, and also the availability of appointments with doctors and other professionals related to the use of the CI [27,67]. However, the findings of the current study did not show any of the above factors as significant influences of the stress experienced by the MCHL. Similar findings were noted in a study by Continisio et al. [6], which reported no correlation between parental stress and the number of children, degree of hearing loss, age of diagnosis, use of hearing devices, and age of rehabilitation. Another study by Piplani et al. [27] also found no correlation between parental stress and child’s age of diagnosis or cochlear implantation.

Intervention at the appropriate time can support and rehabilitate the child and train their family members to cope with difficulties both emotionally and socially, thus improving the psychological health of mothers [68]. The median age of hearing loss identification in this study was 6.5 months and the median age of rehabilitation was 19 months. All of the children in this study were reported to use appropriate amplification devices, with a majority of about 77% using either HA in one ear and CI in the other or unilateral CI. Prakash et al. [28] stated that mothers of children with their hearing amplified with a CI experienced lesser stress and anxiety as their children were able to adapt to their environment. Piplani et al. [27] indicated a positive relationship between the outcomes of the CI and the stress level of the parents. They indicated that the parents were satisfied with the communication skills and educational abilities of their children after increasing the duration of CI use. As mentioned earlier, all but four mothers in the MCHL group indicated that they communicated with their child verbally. Though communication skills were not formally assessed in the present study, it can be inferred from the reported mode of verbal communication with the child that the MCHL were probably satisfied with their child’s communication skills. Thus, it appears that if the parents are happy with the outcome of their child’s rehabilitation, all other factors may not play a major role in parental stress. However, the satisfaction of MCHL with their child’s rehabilitation was not assessed in this study. Therefore, cautious interpretation is necessary, as there may have been mothers with varying degrees of satisfaction with the rehabilitation outcomes and progress of their CHL.

The dynamics of parental stress, learned helplessness, and perceived social support may change depending on the resources, welfare, and financial situations. Most of the CHL in the present study had obtained hearing aids and/or cochlear implants through government-funded schemes. Thus, some financial aid was available to the parents in procuring hearing devices to rehabilitate their CHL. In the present study, children who were attending school attended normal schools and thus had the same opportunities regarding choices and education. However, the educational support that they obtained in school was not explored.

### 4.4. Limitations of the Study

This study has certain limitations. The age of the mother and the gender of the child in the present study are not known. Hence, the influence of these variables on parental stress could not be assessed. It is also not known whether the MCHL who were homemakers were not working even before their child was born or they quit their job because of their child’s hearing loss. This can provide additional insights into the psychological well-being of the MCHL. The children in the MTDC group were significantly younger than in the MCHL group, and a higher percentage of parents had higher education. The MCHL group is heterogeneous in terms of the children’s degree of hearing loss, and this may have modified the severity of parental stress in this group.

### 4.5. Conclusions

Using questionnaires, we assessed, compared, and examined the relationship between the level of parental stress, learned helplessness, and perceived social support among MCHL and MTDC. The findings indicate that the MCHL and MTDC did not vary significantly with regard to parameters such as parental stress and learned helplessness. However, the perceived social support from family and friends was lower for MCHL than MTDC. Also, perceived social support was significantly negatively correlated with learned helplessness and parental stress. Thus, there is a greater need to provide social support to MCHL. Family members and friends should be actively involved in counseling as a part of rehabilitation and therapy to improve the quality of care for CHL and improve the quality of life of MCHL.

## Figures and Tables

**Table 1:** Age, number of children, birth order, and type of family of children with hearing loss and typically developing children.

Variables		Children with Hearing Loss	Typically Developing Children
Age (months)	Mean (SD)	61.14 (30.28)	42.86 (27.26)
	Median	56	36
	Range	12–149	12–146
No. of children	One child	46	51
	More than one child	54	39
Birth order	First	58	69
	Second	34	17
	Third	7	2
	Other	1	2
Type of family *	Nuclear	53	58
	Joint	47	32

* Type of family was considered nuclear when only child with hearing loss lived with its parents and siblings, while it was considered a joint family when any other family member lived with the child with hearing loss, such as grandparents.

**Table 2:** Education and occupation of mothers and fathers of children with hearing loss and typically developing children.

	Parents of Children with Hearing Impairment		Parents of Typically Developing Children	
	Mother N (%)	Father N (%)	Mother N (%)	Father N (%)
**1. Education**				
Profession or Honors (e.g., Doctors, Dentists, Ph.D.)	1 (1%)	0 (0%)	3 (3.33%)	3 (3.33%)
Postgraduate (Master’s or equivalent)	11 (11%)	8 (8%)	18 (20%)	18 (20%)
Graduate (Bachelor’s or equivalent)	43 (43%)	34 (34%)	51 (56.67%)	39 (43.33%)
Plus 2 or diploma (senior or upper secondary)	30 (30%)	30 (30%)	17 (18.89%)	21 (23.33%)
10th grade or less (Secondary or less)	15 (15%)	28 (28%)	1 (1.11%)	9 (10%)
**2. Occupation**				
Legislators, senior officials and managers, income tax officer	0 (0%)	0 (0%)	2 (2.22%)	7 (7.78%)
Professionals	3 (3%)	4 (4%)	20 (22.22%)	11 (12.22%)
Technicians and associate professionals	1 (1%)	15 (15%)	8 (8.89%)	34 (37.78%)
Clerks, skilled workers and shop and market sales workers	5 (5%)	42 (42%)	4 (4.44%)	25 (27.78%)
Agricultural and fishery workers.	0 (0%)	3 (3%)	0 (0%)	1 (1.11%)
Business/self-employed	0 (0%)	19 (19%)	0 (0%)	5 (5.56%)
Unemployed	91 (91%)	4 (4%)	56 (62.22%)	0 (0%)
Could not ascertain/retired/not mentioned	0 (0%)	13 (13%)	0 (0%)	7 (7.78%)

**Table 3:** Mean, standard deviation, range, and the results of the independent samples t-test of the scores of the parental stress scale, learned helplessness scale and PSS-Fa, PSS-Fr Scale, and PSS-Total score in the MTDC and MCHL groups.

Questionnaires	Mean (SD)	Range	t (df)	*p*-Value
Parental Stress Scale				
MTDC	33.01 (8.09)	21–56	−1.42 (188)	0.16
MCHL	34.55 (6.89)	21–52		
Learned helplessness scale				
MTDC	45.28 (5.68)	30–59	−1.29 (188)	0.20
MCHL	46.28 (5.04)	32–60		
Perceived Social Support-Friends				
MTDC	11.31 (3.61)	1–19	7.25 (188)	<0.001 ^1^
MCHL	7.04 (4.42)	0–17		
Perceived Social Support-Family				
MTDC	13.56 (3.65)	2–20	4.52 (188)	<0.001 ^1^
MCHL	10.94 (4.26)	0–18		
Perceived Social Support-Total				
MTDC	24.87 (6.18)	8–39	7.03 (188)	<0.001 ^1^
MCHL	17.98 (7.21)	0–34		

Note. MTDC: mothers of typically developing children; MCHL: mothers of children with hearing loss; ^1^ *p* < 0.05 is statistically significant

## Data Availability

The original contributions presented in this study are included in the Supplementary Materials; further inquiries can be directed to the corresponding author.

## Supplementary Materials

The following supporting information can be downloaded at: https://www.mdpi.com/article/10.3390/audiolres15010001/s1.
